# Physical and cognitive doping in university students using the unrelated question model (UQM): Assessing the influence of the probability of receiving the sensitive question on prevalence estimation

**DOI:** 10.1371/journal.pone.0197270

**Published:** 2018-05-15

**Authors:** Pavel Dietz, Anne Quermann, Mireille Nicoline Maria van Poppel, Heiko Striegel, Hannes Schröter, Rolf Ulrich, Perikles Simon

**Affiliations:** 1 Research Group Physical Activity and Public Health, Institute of Sports Science, University of Graz, Graz, Austria; 2 Working Group Social and Health Sciences of Sport, Institute for Sports and Sports Science, Karlsruhe Institute of Technology, Karlsruhe, Germany; 3 Department of Sports Medicine, Rehabilitation and Disease Prevention, Institute of Sports Science, University of Mainz, Mainz, Germany; 4 Institute of Exercise and Public Health, Faculty of Sport Science, University of Leipzig, Leipzig, Germany; 5 Department of Sports Medicine, University of Tuebingen, Tuebingen, Germany; 6 German Institute for Adult Education, Bonn, Germany; 7 Department of Psychology, University of Tuebingen, Tuebingen, Germany; Rutgers The State University of New Jersey, UNITED STATES

## Abstract

**Study objectives:**

In order to increase the value of randomized response techniques (RRTs) as tools for studying sensitive issues, the present study investigated whether the prevalence estimate for a sensitive item ${\hat{\pi }}_{s}$ assessed with the unrelated questionnaire method (UQM) is influenced by changing the probability of receiving the sensitive question *p*.

**Material and methods:**

A short paper-and-pencil questionnaire was distributed to 1.243 university students assessing the 12-month prevalence of physical and cognitive doping using two versions of the UQM with different probabilities for receiving the sensitive question (*p* ≈ 1/3 and *p* ≈ 2/3). Likelihood ratio tests were used to assess whether the prevalence estimates for physical and cognitive doping differed significantly between *p* ≈ 1/3 and *p* ≈ 2/3. The order of questions (physical doping and cognitive doping) as well as the probability of receiving the sensitive question (*p* ≈ 1/3 or *p* ≈ 2/3) were counterbalanced across participants. Statistical power analyses were performed to determine sample size.

**Results:**

The prevalence estimate for physical doping with *p* ≈ 1/3 was 22.5% (95% CI: 10.8–34.1), and 12.8% (95% CI: 7.6–18.0) with *p* ≈ 2/3. For cognitive doping with *p* ≈ 1/3, the estimated prevalence was 22.5% (95% CI: 11.0–34.1), whereas it was 18.0% (95% CI: 12.5–23.5) with *p* ≈ 2/3. Likelihood-ratio tests revealed that prevalence estimates for both physical and cognitive doping, respectively, did not differ significantly under *p* ≈ 1/3 and *p* ≈ 2/3 (physical doping: *χ*^*2*^ = 2.25, *df* = 1, *p* = 0.13; cognitive doping: *χ*^*2*^ = 0.49, *df* = 1, *p* = 0.48). Bayes factors computed with the Savage-Dickey method favored the null (“the prevalence estimates are identical under *p* ≈ 1/3 and *p* ≈ 2/3”) over the alternative (“the prevalence estimates differ under *p* ≈ 1/3 and *p* ≈ 2/3”) hypothesis for both physical doping (BF = 2.3) and cognitive doping (BF = 5.3).

**Conclusion:**

The present results suggest that prevalence estimates for physical and cognitive doping assessed by the UQM are largely unaffected by the probability for receiving the sensitive question *p*.

## Introduction

### Socially sensitive research

Whenever studies with the aim to assess the prevalence rates of *socially sensitive issues* are performed, it is a challenge for researchers to measure these rates validly [1, 2]. This challenge starts by giving a precise definition of the term “sensitive” research. According to Sieber & Stanley [3], *socially sensitive research* is defined as “studies in which there are potential consequences or implications, either directly for the participants in the research or for the class of individuals represented by the research”. Lee & Renzetti [1] and Lee [4] described it as research which potentially poses a substantial *threat* for those who are or have been involved in it. As a consequence, when sensitive topics are studied, participants often react in a way that negatively affects the validity of study results (underreporting and non-responding) due to hesitating to provide compromising information about themselves [5, 6]. Examples for socially sensitive topics are domestic violence [7], political activism [8], homicide and rape [9], mental health [10], death, murder and abortion [11, 12], traumatic childbirth [13], and sexual health [14], according to Fahie [15]. Other examples that demonstrate how common sensitive issues are even in every-day aspects are medical adherence [16], attitudes towards foreigners [17], or cooperation in social interactions [18]. Another topic which has been considered to be socially sensitive is the use of prohibited substances for enhancing physical performance in athletes (doping) [19–21] and the use of illicit and prescription drugs for enhancing cognitive performance (pharmacological neuroenhancement) in students, academics, and workers [22–24].

### Randomized response designs

Warner [25, 26] stated that participants may be more willing to reveal sensitive information if participant’s anonymity was guaranteed and introduced the first *randomized response design*, the so called Warner’s original method. Randomized response designs (also called randomized response technique; RRT) are developed specifically to obtain more valid estimates when sensitive topics are studied through guaranteeing a maximum amount of anonymity [27, 28]. Beside Warner’s original method, several further RRTs have been developed such as the *unrelated question method* (UQM) [29], the *forced response method* [30, 31], the *item count technique *[32], the *crosswise method* [33], the *cheater detection model* (CDM) [34], and the *stochastic lie detector* [35].

In their meta-analysis of 38 randomized response validation studies, Lensvelt-Mulders et al. [28] concluded that RRTs yield more valid results when assessing socially sensitive items compared to more conventional survey techniques such as face-to-face interviewing, self-administered questionnaires, and telephone interviewing (see also Moshagen et al. [36] for a recent discussion on the validation of questioning techniques assessing sensitive issues). Furthermore, they stated that although RRT results are more valid compared to results of conventional survey techniques, there is still room for improvement and the more efficient RRTs become, the larger will be their value as a tool to study sensitive topics. For example, a recent study by Hoffmann et al. [37] indicated that although the mean perceived privacy protection of four chosen RRT variants was higher compared to direct questioning, the mean perceived privacy protection varied between these four variants.

### The unrelated question method (UQM)

**Fig 1:**
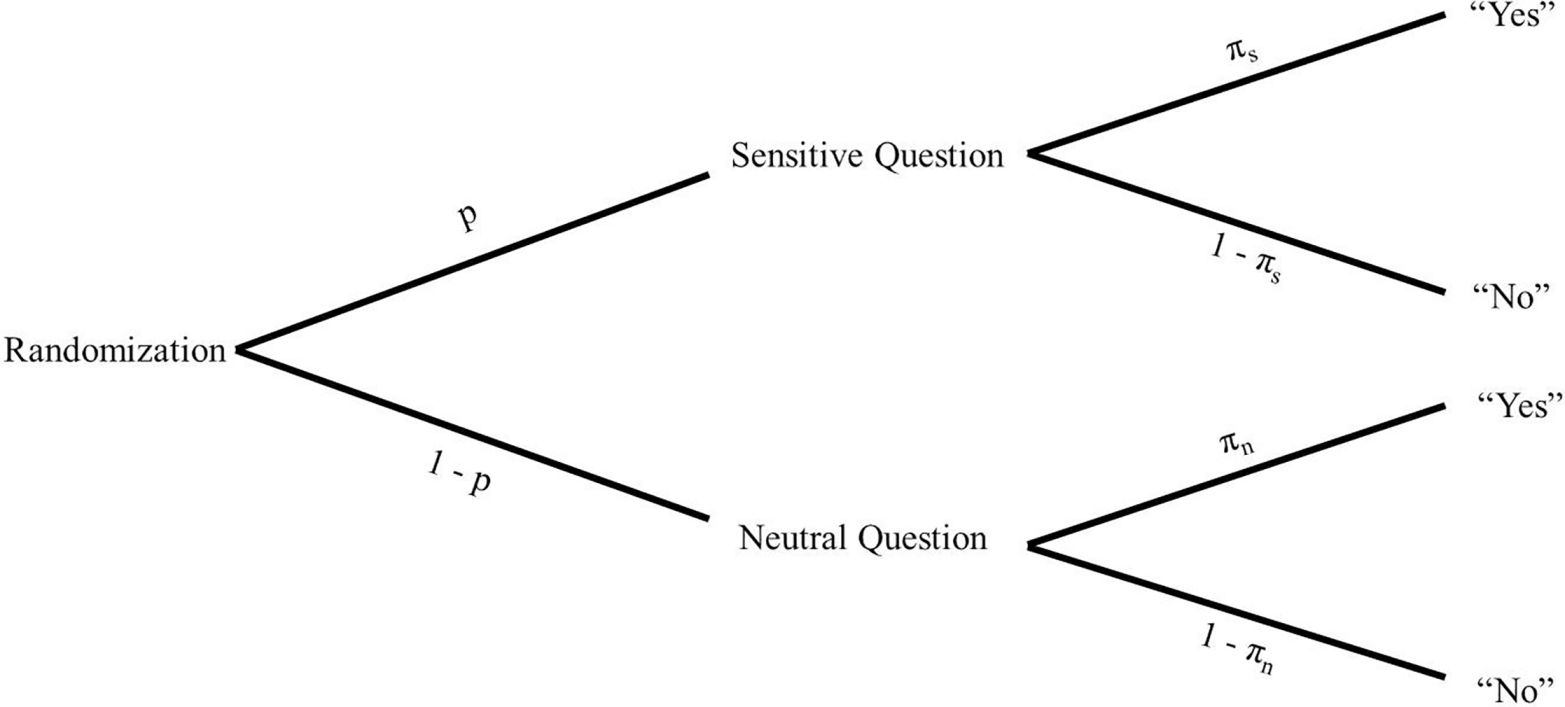
Tree diagram of the unrelated question method (UQM). *p* denotes the probability of receiving the sensitive question; *π*_*n*_ denotes the probability of answering the neutral question with “yes”; *π_s_* denotes the prevalence for the sensitive question.

Within the present article, we focus on the UQM [22–24, 29]. Using the UQM, each participant is guided with the aid of a randomizer (dice, coin, or deck of cards) to one of two questions, which should be answered honestly: a neutral (non-sensitive) question (A) or a sensitive question (assessing the sensitive item; B). The probability of receiving the sensitive question (based on the randomization) is denoted as *p* and the probability of receiving the neutral question as *1-p* (Fig 1). For example, in a former study [20, 21] participants were asked to draw a card of their choice from a deck of 20 cards. 15 cards contained the sensitive question and 5 cards the neutral question. Thus, the probability of receiving the sensitive question *p* was 3/4 and the probability of receiving the neutral question *1-p* was 1/4. The neutral question has to fulfill the criteria to get answered by a sample with a certain probability with “yes”. This probability for answering the neutral question with “yes” is denoted as *π*_*n*_ and is known by the interviewers. Only the participant knows the outcome of the randomization process. Thus, the specific statement the participant answers with “yes” or no” is hidden from the experimenter, thereby providing both objective and subjective anonymity. However, based on the proportion of total “yes” responses of a sample (denoted as *a*) and given that the probabilities *π*_*n*_ and *p* are known by the researchers, a prevalence estimate for the sensitive question ${\hat{\pi }}_{s}$ can be calculated using the formula ${\hat{\pi }}_{S}=\frac{a-(1-p)\cdot \pi_{n}}{p},$ and a 95% confidence interval (CI) for the unknown prevalence estimate can be used on the basis of the sampling variance where n denotes the sample size: $Var({\hat{\pi }}_{S})=\frac{a\cdot (1-a)}{N\cdot p^{2}}$.

According to Lensvelt-Mulders et al. [28] the UQM has been rated to be one of the most efficient designs for measuring socially sensitive issues compared to other RRTs. In order to follow their recommendation to increase the efficacy of RRTs, Dietz et al. [22] developed a paper-and-pencil version of the UQM that enabled the researchers to randomly guide the participants to either answer the neutral or the sensitive question without using a randomization device such as dice, coin or deck of cards. This improvement enabled researchers to assess higher case numbers in less time. In addition, Schröter et al. [38] used the UQM and a CDM to assess the prevalences for physical and cognitive doping in triathletes and observed no meaningful differences for the prevalences estimated with CDM and UQM.

### Aim of the present study

In order to increase the value of RRTs as a tool for studying sensitive topics, as suggested by Lensvelt-Mulders et al. [28], the present study investigated whether the prevalence estimate ${\hat{\pi }}_{s}$ of the UQM is influenced by changing the probability of receiving the sensitive question *p*. In other words, does the UQM generate different prevalence estimates for a sensitive issue when different *p*s are used?

For example, a major concern is that some participants may hesitate to respond truthfully with “yes” to the sensitive question because an affirmative response leaves open the possibility that the respondent has the stigmatizing attribute; in order to avoid this impression, a respondent may provide a dishonest “no” response instead. Specifically, according to Bayes’s rule, participants should become increasingly reluctant to provide a “yes” response to the sensitive question when *p* increases toward one [39] and will therefore cheat more. This is because the conditional probability P(*A*|“Yes”) of having the stigmatizing attribute *A* given a “Yes” response increases with probability *p* (see S1 Appendix). Accordingly, we expect smaller prevalence estimates for *p* ≈ 2/3 than for *p* ≈ 1/3. Note that small values of *p* produce relatively large standard errors of the prevalence estimate unless especially large samples sizes are used. Therefore, *p* ≈ 2/3 is more feasible than *p* ≈ 1/3. For this reason, we assessed the prevalence estimates of physical and cognitive doping in a sample of university students using two UQM versions with different probabilities of receiving the sensitive question (*p* ≈ 1/3 and *p* ≈ 2/3). According to Dietz et al. [19], the term *physical doping* describes “the intake of illicit or banned substances to improve physical performance” (e.g. anabolic androgenic steroids, human growth hormones, erythropoietin) and the term *cognitive doping* includes “illicit drugs (e.g. cocaine) and prescription drugs (…) such as stimulants (e.g. methylphenidate and amphetamines), antidepressants, beta-blockers, or modafinil” with the aim to improve cognitive functions such as memory, attention, learning performance, or mood. Both types of substances have been reported by previous studies to be frequently used by students [40–45] and therefore, the collective of university students seems to be highly suitable for testing our hypothesis.

## Material and methods

### Survey procedure

On the basis of a previous performed survey concerning performance enhancing substances in university students using the RRT [22], a short paper-and-pencil questionnaire was distributed among university students of the University of Mainz, Germany at the beginning of classes. In order to be able to recruit an approximate representative sample of students based on age, sex, and field of study, all major classes of the different disciplines were identified using the online study administration platform of the university. Two weeks before the survey was performed (the survey was performed in the first third of the semester in order to reach a high number of students), all teachers/lecturers were informed about the survey by email. Once the questionnaire was distributed by our researcher team at the beginning of the classes, a briefed assistant introduced the students to the survey procedure and stressed the anonymity of the RRT. Students were told to fill-in the questionnaire immediately at the beginning of the class and to drop it into black boxes by the classrooms doors at the end of the class.

### Questionnaire

At the beginning of the single-paged paper-and-pencil questionnaire, a short introduction was given explaining the aim of the study and guaranteeing the anonymity of the survey. Afterwards the terms ‘physical doping’ and ‘cognitive doping’ (in German, the term “Doping” is commonly used for physical doping whereas the term ‘Hirndoping’, which literally translates to brain doping, is commonly used for cognitive doping) were described to the participants:

“Substances for physical and cognitive enhancement (doping and brain doping) are pharmaceuticals or illicit drugs, which you cannot buy in a drug store and that were not prescribed to you to treat a disease. The only reason why you use this substance is to reach a certain goal.

Physical Doping: The goal is to improve physical performance or to improve your look. Examples: anabolic steroids (e.g. nandrolone, testosterone), peptide hormones (e.g. erythropoietin, growth hormones), stimulants (e.g. amphetamines).

Cognitive doping: The goal is to improve cognitive performance such as attention, alertness, and mood. Examples: stimulants (e.g. amphetamines), caffeine tablets, cocaine, Ritalin^®^ (methylphenidate), mephedrone (coffee and tea do not count to these substances).

After the introduction and the description of the terminology, two RRT questions followed using the UQM; one question to assess the 12-month prevalence for physical doping, and one to assess the 12-month prevalence of cognitive doping. For this study, we adapted the questions used in previous studies using the UQM [19, 22, 23, 38, 46] but used two different probabilities of receiving the sensitive question. One half of the questions contained a probability of receiving the sensitive question of *p* ≈ 1/3 and the other half of *p* ≈ 2/3. For example, to assess the prevalence of cognitive doping by using a *p* ≈ 2/3, the following text appeared in the questionnaire:

___________________________________________________________________________

Please consider a certain birthday (yours, your mother`s, etc.). Is this birthday in the first third of a month (1^st^ to 10^th^ day)? If “Yes”, please proceed to question A; if “No”, please proceed to question B.

Question A: Is this birthday in the first half of the year (prior to the 1^st^ of July)?

Question B: Did you use brain-doping substances during the last 12 months?

Your answer to question A or question B is (note that only you know which of the two questions you will answer):

YES NO

___________________________________________________________________________

Thus, 32.9% (120 of 365.25) of the students received the non-sensitive question A, whereas 67.1% ≈ 2/3 (245.25 of 365.25) receive the sensitive question B.

To assess, for example, the prevalence of physical doping by using a *p* ≈ 1/3, the following text appeared in the questionnaire:

___________________________________________________________________________

Please consider a certain birthday (yours, your mother`s, etc.) Is this birthday in the first two thirds of a month (1^st^ to 20^th^ day)? If “Yes”, please proceed to question A; if “No”, please proceed to question B.

Question A: Is this birthday in the first half of the year (prior to the 1^st^ of July)?

Question B: Did you use doping substances during the last 12 months?

Your answer to question A or question B is (note that only you know which of the two questions you will answer):

YES NO

___________________________________________________________________________

Thus, 65.7% (240 of 365.25) of the students received the non-sensitive question A, whereas 34.3% ≈ 1/3 (125.25 of 365.25) receive the sensitive question B. The probability of answering the non-sensitive question with ‘yes’ is *π*_*n*_ = 49.7 (181.25 of 365.25 ≈ 1/2) for each question. For calculating the prevalence estimates and confidence intervals the exact (non-rounded) probabilities for *p* and *π*_*n*_ were used.

In order to avoid a possible influence of the question order on the results, the order of questions (physical doping and cognitive doping) as well as the probabilities of receiving the sensitive question (*p* ≈ 1/3 or *p* ≈ 2/3) were counterbalanced across participants. As a consequence, eight different versions of the questionnaire were created (Table 1). At the end of the questionnaire, four characteristics of the participants were asked. These were gender (female/male), age (metric), number of semester (metric), and field of study (nominal).

**Table 1:** Explanation of the different questionnaire versions.

Version	First sensitive question	*p* of first question	Second sensitive question	*p* of second questions
1	BD	2/3	D	1/3
2	BD	1/3	D	1/3
3	BD	2/3	D	2/3
4	BD	1/3	D	2/3
5	D	2/3	BD	1/3
6	D	1/3	BD	1/3
7	D	2/3	BD	2/3
8	D	1/3	BD	2/3

BD, brain doping; D, doping; *p*, probability of receiving the sensitive question

### Statistics

Statistical power analyses [47] were performed to determine the necessary sample size for detecting an overall prevalence of 15% for physical and cognitive doping respectively, which, according to the literature [19, 22, 40], appears to be a lower conservative value of the prevalences. The null hypothesis of this power analysis assumes that *π_s_* = 0. Under this assumption, for the questions using *p* ≈ 1/3, a sample size of 550 provides a power of approximately 0.8 for rejecting the null hypothesis and for the questions using *p* ≈ 2/3, a sample size of 150 provides a power of approximately 0.9 for rejecting the null hypothesis. This difference is based on the circumstance that the higher *p* is, the more people of a certain sample receive the sensitive question. Since approximately every second question contained *p* ≈ 1/3 (Table 1), a total sample size of 1,100 valid questionnaires was needed.

A similar approach as introduced in [47] was used to conduct a power analysis for detecting a significant difference between the prevalence estimates under the two probability conditions. Specifically, based on former studies with *p* ≈ 2/3, we proceeded from a prevalence estimate of 0.15 for this probability condition. For *p* ≈ 1/3, however, one might assume that participants feel more protected than with *p* ≈ 2/3. Thus, one may assume a prevalence estimate of 0.20, 0.25, or 0.30 under *p* ≈ 1/3. If *n* = 600 participants are tested under each probability condition, one computes a statistical power of 0.33, 0.78, or 0.98, respectively, for detecting a significant difference between the prevalence estimates in the two probability conditions.

Descriptive data are presented as mean ± SD values and were calculated using SPSS software, version 22. Prevalence estimates ${\hat{\pi }}_{s}$ for physical doping (separately for *p* ≈ 1/3 and *p* ≈ 2/3), and cognitive doping (separately for *p* ≈ 1/3 and *p* ≈ 2/3) are presented as percentages with 95% confidence intervals (CI) and standard error (SE). MatLab version R2015a was used to calculate a combined prevalence estimate (*p* ≈ 1/3 and *p* ≈ 2/3) for physical doping and cognitive doping. Moreover, a likelihood ratio test was used to assess whether the prevalence estimates for the two *p*s differ significantly. Finally, the Savage-Dickey method [48] was used to compute the Bayes factor B01 for H_0_ (i.e. the prevalence estimates are identical under *p* ≈ 1/3 and *p* ≈ 2/3) versus H_1_ (i.e. the prevalence estimates differ under *p* ≈ 1/3 and *p* ≈ 2/3). This Bayesian analysis treated the UQM as a multinomial processing tree. Markov chain Monte Carlo sampling with the software RJAGS [49] was then employed to compute the posterior distribution of the difference between the prevalence estimate for *p* ≈ 1/3 and the prevalence estimate for *p* ≈ 2/3 under H_0_ as well as under H_1_. The Savage-Dickey density ratio was calculated at zero difference by using a kernel density estimator. This Bayesian analysis was performed under R (see Thielmann et al. [50] for a similar approach using a different RRT model).

Ethical approval to conduct this study was obtained by the Ethics Committee of the Medical Faculty and the University Medical Center of the University of Tuebingen, Germany (project number 095/2011BO2).

## Results

Of the 1,243 questionnaires distributed at the beginning of the classes, 1,206 questionnaires were returned, resulting in a response rate of 97%. The distribution of the eight questionnaire versions (Table 1) is presented within Table 2. Of the 1,206 participants that returned a questionnaire, 33 participants (2.7%) did not fill in any of the two RRT questions and nine participants (0.7%) filled in the first RRT question only. In summary, 1,169 participants answered to the RRT question concerning physical doping (*p* ≈ 1/3 or *p* ≈ 2/3) and 1,168 participants concerning cognitive doping (*p* ≈ 1/3 or *p* ≈ 2/3). The characteristics of the respondents are presented within Table 3.

**Table 2:** Distribution of the different questionnaire versions.

Version	*N*	Percentage (%)
1	154	12.8
2	149	12.4
3	159	13.2
4	153	12.7
5	151	12.5
6	150	12.4
7	153	12.7
8	137	11.4

**Table 3:** Characteristics of the respondents.

Variable	Value
**Gender**, no (%)	
Female	675 (42.1)
Male	491 (57.9)
**Age**, range, yrs (mean ± SD)	16–64 (21.9 ± 3.1)
**Semester**, range (mean ± SD)	1–20 (3.3 ± 2.2)
**Field of study**^**#**^, no (%)	
Economics or law	376 (32.4)
Medicine, psychology, or natural sciences	242 (20.9)
Languages or education	235 (20.3)
Culture sciences	224 (19.3)
Sports science	81 (7.0)

^#^The items for the variable field of study were grouped on the basis of a previous study [22]

The prevalence estimate for physical doping with *p* ≈ 1/3 was 22.5% (95% CI: 10.8–34.1), and with *p* ≈ 2/3 it was 12.8% (95% CI: 7.6–18.0). A likelihood-ratio test revealed no significant difference between these two estimates, *χ*^*2*^ = 2.25, *df* = 1, *p* = 0.134. For cognitive doping with *p* ≈ 1/3, the estimated prevalence was 22.5% (95% CI: 11.0–34.1), whereas it was 18.0% (95% CI: 12.5–23.5) with *p* ≈ 2/3. Again, these two estimates did not significantly differ, *χ*^*2*^ = 0.49, *df* = 1, *p* = 0.482. Combined physical doping prevalence (*p* ≈ 1/3 and *p* ≈ 2/3) and combined cognitive doping prevalence was 14.4% (95% CI: 9.6–19.2; SE: 2.5) and 18.8% (95% CI: 13.8–23.8; SE: 2.5), respectively (Table 4). The Bayesian analysis revealed Bayes factors of 2.3 and 5.3 for physical and cognitive doping, respectively. According to these values (corresponding to “weak” and “positive” evidence, respectively; see Raftery [51] and Wagenmakers [52]), the null hypotheses (i.e. the prevalence estimates are identical under *p* ≈ 1/3 and *p* ≈ 2/3) should be favored over the alternative hypothesis (i.e. the prevalence estimates differ under *p* ≈ 1/3 and *p* ≈ 2/3).

**Table 4:** Estimated 12-month prevalences for physical and cognitive doping using the UQM for *p* ≈ 1/3 and *p* ≈ 2/3*.

Variable	‘yes’	‘no’	*a*	${\hat{\pi }}_{s}(\%)$	$\mathrm{SE}({\hat{\pi }}_{s})$	95% CI
Physical doping *p* ≈ 1/3	233	345	0.403	22.5	5.9	10.8–34.1
Physical doping *p* ≈ 2/3	147	444	0.249	12.8	2.6	7.6–18.0
Cognitive doping *p* ≈ 1/3	238	352	0.403	22.5	5.9	11.0–34.1
Cognitive doping *p* ≈ 2/3	164	414	0.284	18.0	2.8	12.5–23.5

*Please note that the accurate probabilities for the two different *p*´s and *π*_*n*_ are given in the methods section above

## Discussion

The aim of this study was to investigate whether the prevalence estimate for a sensitive item assessed with the unrelated questionnaire method (UQM) is influenced by changing the probability of receiving the sensitive question *p*. This was done by assessing the 12-month prevalence estimates for physical doping and cognitive doping in a collective of university students using two different probabilities of receiving the sensitive question (*p* ≈ 1/3 and *p* ≈ 2/3). Therefore, the study design from a previously performed study in university students was adapted [22]. Similar to the previous study, the present study showed a high response rate of more than 90%. In order to evaluate representativeness of a surveyed sample, Baruch [53] stated that not only the rate of responded questionnaires but also the rate of useable (valid) questionnaires is important. Since only 33 participants who returned a questionnaire did not provide answers to any of the RRT questions, the rate of usable questionnaires was 94.4%, which is an excellent percentage compared to other surveys addressing the use of substances in student collectives [42, 54–56].

Likelihood ratio tests revealed that the prevalence estimates for physical doping (22.5% and 12.8%) as well as for cognitive doping (22.5% and 18.0%) estimated with *p* ≈ 1/3 and *p* ≈ 2/3 were not significantly different. In addition, a Bayes analysis revealed fairly more support for the null hypothesis (“prevalence estimates are identical for *p* ≈ 1/3 and *p* ≈ 2/3”) than for the alternative hypothesis (“prevalence estimates differ for *p* ≈ 1/3 and *p* ≈ 2/3”). Consequently, these results provide no support for our initial assumption that according to Bayes’s rule, participants should become increasingly reluctant to provide a “yes” response to the sensitive question when *p* increases toward one (see S1 Appendix). Contrary, the prevalence estimation by UQM seems to be rather robust against a manipulation of the probability *p* of receiving the sensitive question. A similar result was recently reported in a study by Hilbig and Zettler (2015, Experiment 5), in which the prevalence of cheating behavior in a coin-toss task was largely unaffected by the randomization probability (i.e., the probability of winning an incentive) [57].

The present findings might suggest that one should implement a high value of *p* when using the UQM in sensitive surveys, because for a fixed *n*, power increases with p [47]. However, such a conclusion might be premature on the sole basis of the present study. First, the objective amount of privacy protection by UQM decreases with increasing *p*. Therefore, it is most likely that also the subjective amount of privacy protection decreases with increasing *p*. The exact relation of felt privacy protection and *p* may be influenced by several factors such as the subjective sensitivity of the sensitive question, the implemented randomizer, or the overall setup of the survey. It could be argued that in the present study, the subjective sensitivity of doping behavior was comparably low for the surveyed students, because different from athletes, doping behavior would have no immediate consequences even in case of revelation. Consistent with this notion, the estimated prevalence rates for doping behavior in the present study were comparable with a previous study surveying university students with the UQM [22] but considerably higher than in a previous study surveying recreations competitive athletes with the UQM [38].

Therefore, it seems necessary to perform further studies assessing individuals’ attitudes and beliefs towards sensitive items and possible interaction effects of subjective sensitivity and privacy protection on prevalence estimation by the UQM. Furthermore, perceived privacy protection of UQM should also be compared with other RRTs, because Hoffmann et al. [36] showed that perceived privacy protection varies between different indirect techniques. Second, it could be argued that–given the higher absolute estimated prevalence rates for *p* ≈ 1/3 than for *p* ≈ 2/3 for both physical and cognitive doping–participants indeed tended to change their response behavior (thus, more dishonest answers for *p* ≈ 2/3 than for *p* ≈ 1/3) but that the power of the present study was not sufficient to detect this difference. Therefore, future studies should increase the sample size to provide a more robust test of a possible decrease of perceived privacy protection with increasing *p* and its resulting effect on prevalence estimation. For example, this might be achieved by further increasing the difference between the two p values of receiving the sensitive question.

## Supporting information

S1 Appendix This appendix shows how this probability depends on the parameters *p*, *π_s_*, and *π_n_*. (DOCX)

S1 Dataset (SAV)

## References

[1] Lee RM, Renzetti CM. The Problems of Researching Sensitive Topics: An Overview and Introduction. Am Behav Sci. 1990;33: 510–528.

[2] Dickson-Swift V, James EL, Kippen S, Liamputtong P. Researching sensitive topics: Qualitative research as emotion work. Qual Res. 2009;9: 61–79.

[3] Sieber JE, Stanley B. Ethical and professional dimensions of socially sensitive research. Am Psychol. 1988; 43: 49–55. 3348539 10.1037//0003-066x.43.1.49

[4] Lee RM. Doing research on sensitive topics. London, Newbury Park, Calif: Sage Publications; 1993.

[5] Sudman S, Bradburn NM. Asking questions. 1st ed San Francisco: Jossey-Bass; 1982. (Jossey-Bass series in social and behavioral sciences).

[6] Rasinski KA, Willis GB, Baldwin AK, Yeh W, Lee L. Methods of data collection, perceptions of risks and losses, and motivation to give truthful answers to sensitive survey questions. Appl Cognit Psychol. 1999;13:465–484.

[7] Enosh G. The interactive construction of narrative styles in sensitive interviews: The case of domestic violence research. Qual Inq. 2005;11: 588–617.

[8] Possick C. Reflexive Positioning in a politically sensitive situation: Dealing with the threats of researching the West Bank settler experience. Qual Inq. 2009;15: 859–875.

[9] Sollund R. Tested Neutrality: Emotional challenges in qualitative interviews on homicide and rape. J Scand Stud Criminol Crime Prev. 2008;9: 181–201.

[10] Mitchell W, Irvine A. I’m okay, you’re okay?: Reflections on the well-being and ethical requirements of researchers and research participants in conducting qualitative fieldwork interviews. Int J Qual Methods. 2008;7: 31–44.

[11] Lipscomb M. Participant overexposure and the role of researcher judgement. Nurse Res. 2010;17: 49–59. doi: 10.7748/nr2010.07.17.4.49.c7924 20712234 10.7748/nr2010.07.17.4.49.c7924

[12] Goodrum S, Keys JL. Reflections on two studies of emotionally sensitive topics: Bereavement from murder and abortion. Int J Soc Res Methodol. 2007;10: 249–258.

[13] Elmir R, Schmied V, Jackson D, Wilkes L. Interviewing people about potentially sensitive topics. Nurse Res. 2011;19: 12–16. doi: 10.7748/nr2011.10.19.1.12.c8766 22128582 10.7748/nr2011.10.19.1.12.c8766

[14] Walls P, Parahoo K, Fleming P, McCaughan E. Issues and considerations when researching sensitive issues with men: examples from a study of men and sexual health. Nurse Res. 2010;18: 26–34. doi: 10.7748/nr2010.10.18.1.26.c8045 21138083 10.7748/nr2010.10.18.1.26.c8045

[15] Fahie D. Doing sensitive research sensitively: Ethical and methodological issues in researching workplace bullying. Int J Qual Methods. 2014;13: 19–36.

[16] Moshagen M, Musch J, Ostapczuk M, Zhao Z. Reducing socially desirable responses in epidemiologic surveys: an extension of the randomized-response technique. Epidemiology 2010;21: 379–382. doi: 10.1097/EDE.0b013e3181d61dbc 20386172 10.1097/EDE.0b013e3181d61dbc

[17] Ostapczuk M, Musch J, Moshagen M. A randomized-response investigation of the education effect in attitudes towards foreigners. Eur J Soc Psychol 2009;39: 920–931.

[18] Moshagen M, Hilbig BE, Musch J. Defection in the dark?: A randomized-response investigation of cooperativeness in social dilemma games. Eur J Soc Psychol 2011;41: 638–644.

[19] Dietz P, Ulrich R, Dalaker R, Striegel H, Franke AG, Lieb K et al Associations between Physical and Cognitive Doping—A Cross-Sectional Study in 2.997 Triathletes. PLoS One. 2013;8: e78702 doi: 10.1371/journal.pone.0078702 24236038 10.1371/journal.pone.0078702 PMC3827233

[20] Striegel H, Ulrich R, Simon P. Randomized response estimates for doping and illicit drug use in elite athletes. Drug Alcohol Depend. 2010;106: 230–232. doi: 10.1016/j.drugalcdep.2009.07.026 19740612 10.1016/j.drugalcdep.2009.07.026

[21] Simon P, Striegel H, Aust F, Dietz K, Ulrich R. Doping in fitness sports: estimated number of unreported cases and individual probability of doping. Addiction. 2006;101: 1640–1644. doi: 10.1111/j.1360-0443.2006.01568.x 17034444 10.1111/j.1360-0443.2006.01568.x

[22] Dietz P, Striegel H, Franke AG, Lieb K, Simon P, Ulrich R. Randomized response estimates for the 12-month prevalence of cognitive-enhancing drug use in university students. Pharmacotherapy. 2013;33: 44–50. doi: 10.1002/phar.1166 23307544 10.1002/phar.1166

[23] Franke AG, Bagusat C, Dietz P, Hoffmann I, Simon P, Ulrich R et al Use of illicit and prescription drugs for cognitive or mood enhancement among surgeons. BMC Med. 2013;11: 102 doi: 10.1186/1741-7015-11-102 23570256 10.1186/1741-7015-11-102 PMC3635891

[24] Dietz P, Soyka M, Franke AG. Pharmacological Neuroenhancement in the Field of Economics-Poll Results from an Online Survey. Front Psychol. 2016;7: 520 doi: 10.3389/fpsyg.2016.00520 27148128 10.3389/fpsyg.2016.00520 PMC4835716

[25] Warner SL. Randomized response: a survey technique for eliminating evasive answer bias. J Am Stat Assoc. 1965;60: 63–66. 12261830

[26] Warner SL. The Linear Randomized Response Model. J Am Stat Assoc. 1971;66: 884.

[27] Lensvelt-Mulders G. J. L. M., Hox JJ, van der Heijden P. G. M., Maas C. J. M. Meta-analysis of randomized response research thirty-five years of validation. Socio Meth Res. 2005;33: 319–348.

[28] Lensvelt-Mulders G. J. L. M., Hox JJ, van der Heijden P. G. M. How to Improve the Efficiency of Randomised Response Designs. Qual Quant. 2005;39: 253–265.

[29] Greenberg BG, Abulela A. L. A., Simmons WR, Horvitz DG. Unrelated Question Randomized Response Model—Theoretical framework. J Am Stat Assoc. 1969;64: 520–536.

[30] Boruch RF. Assuring confidentiality of responses in social research: A note on strategies. Am Sociol. 1971;6: 308–311.

[31] Edgell SE, Himmelfarb S, Duchan KL. Validity of forced responses in a randomized response model. Socio Meth Res. 1982;11: 89–100.

[32] Dalton DR, Wimbush JC, Daily CM. Using the Unmatched Count Technique (UCT) to estimate base rates for sensitive behavior. Pers Psychol. 1994;47: 817–829.

[33] Yu J-W, Tian G-L, Tang M-L. Two new models for survey sampling with sensitive characteristic: Design and analysis. Metrika. 2008;67: 251–263.

[34] Pitsch W, Emrich E, Klein M. Doping in elite sports in Germany: results of a www survey. EJSS. 2007;4: 89–102.

[35] Moshagen M, Musch J, Erdfelder E. A stochastic lie detector. Behav Res Methods. 2012;44: 222–31. doi: 10.3758/s13428-011-0144-2 21858604 10.3758/s13428-011-0144-2

[36] Moshagen M, Hilbig BE, Erdfelder E, Moritz A. An experimental validation method for questioning techniques that assess sensitive issues. Exp Psychol. 2014;61: 48–54. doi: 10.1027/1618-3169/a000226 23948389 10.1027/1618-3169/a000226

[37] Hoffmann A, Waubert de Puiseau B, Schmidt A F, Musch J. On the comprehensibility and perceived privacy protection of indirect questioning techniques. Behav Res Methods. 2017;49: 1470–1483. doi: 10.3758/s13428-016-0804-3 27631988 10.3758/s13428-016-0804-3

[38] Schroter H, Studzinski B, Dietz P, Ulrich R, Striegel H, Simon P. A comparison of the Cheater Detection and the Unrelated Question Models: A randomized response survey on physical and cognitive doping in recreational triathletes. PLoS One. 2016;11: e0155765 doi: 10.1371/journal.pone.0155765 27218830 10.1371/journal.pone.0155765 PMC4878800

[39] Fox JA, Tracy PE. Randomized response: A method for sensitive surveys. Beverly Hills, Calif: Sage Publications; 1986.

[40] DeSantis AD, Webb EM, Noar SM. Illicit use of prescription ADHD medications on a college campus: a multimethodological approach. J Am Coll Health. 2008;57: 315–324. doi: 10.3200/JACH.57.3.315-324 18980888 10.3200/JACH.57.3.315-324

[41] Hall KM, Irwin MM, Bowman KA, Frankenberger W, Jewett DC. Illicit use of prescribed stimulant medication among college students. J Am Coll Health. 2005;53: 167–174. doi: 10.3200/JACH.53.4.167-174 15663065 10.3200/JACH.53.4.167-174

[42] McCabe SE, Knight JR, Teter CJ, Wechsler H. Non-medical use of prescription stimulants among US college students: prevalence and correlates from a national survey. Addiction. 2005;100: 96–106. doi: 10.1111/j.1360-0443.2005.00944.x 15598197 10.1111/j.1360-0443.2005.00944.x

[43] Teter CJ, McCabe SE, Boyd CJ, Guthrie SK. Illicit methylphenidate use in an undergraduate student sample: prevalence and risk factors. Pharmacotherapy. 2003;23: 609–617. 12741435 10.1592/phco.23.5.609.34187

[44] Bassoli L, Boncinelli S, Brizzi L, Curci R, Signorelli D, Pazardjiklian I et al Survey of physical activity and doping in a sample of 6,915 students aged 14–18 years. Minerva Pediatr. 2004;56: 317–326. 15252380

[45] Yesalis CE. Use of steroids for self-enhancement: an epidemiologic/societal perspective. AIDS Read. 2001;11: 157–160. 17004353

[46] Dietz P, Dalaker R, Letzel S, Ulrich R, Simon P. Analgesics use in competitive triathletes: its relationship to doping and on predicting its usage. J Sports Sci. 2016;34: 1965–1969. doi: 10.1080/02640414.2016.1149214 26911564 10.1080/02640414.2016.1149214

[47] Ulrich R, Schroter H, Striegel H, Simon P. Asking sensitive questions: A statistical power analysis of randomized response models. Psychol Methods. 2012;17: 623–641. doi: 10.1037/a0029314 22924599 10.1037/a0029314

[48] Wagenmakers E-J, Lodewyckx T, Kuriyal H, Grasman R. Bayesian hypothesis testing for psychologists: a tutorial on the Savage-Dickey method. Cogn Psychol. 2010;60:158–189. doi: 10.1016/j.cogpsych.2009.12.001 20064637 10.1016/j.cogpsych.2009.12.001

[49] Plummer M. rjags: Bayesian graphical models using MCMC [Computer software manual]. (R package version 4–6). 2016.

[50] Thielmann I, Heck DW, Hilbig BE. Anonymity and incentives: An investigation of techniques to reduce socially desirable responding in the Trust Game. Judgment and Decision Making 2016;11: 527–536.

[51] Raftery AE. Bayes Factors and BIC—Comment on "A Critique of the Bayesian Information Criterion for Model Selection". Sociol Methods Res 1999;27: 411–427.

[52] Wagenmakers E-J. A practical solution to the pervasive problems of *p* values. Psychon Bull Rev 2007;14: 779–804 18087943 10.3758/bf03194105

[53] Baruch Y. Response rate in academic studies—a comparative analysis. Hum Relat. 1999;52: 421–538.

[54] Franke AG, Christmann M, Bonertz C, Fellgiebel A, Huss M, Lieb K. Use of coffee, caffeinated drinks and caffeine tablets for cognitive enhancement in pupils and students in Germany. Pharmacopsychiatry. 2011;44: 331–338. doi: 10.1055/s-0031-1286347 21993866 10.1055/s-0031-1286347

[55] Franke AG, Bonertz C, Christmann M, Huss M, Fellgiebel A, Hildt E et al Non-medical use of prescription stimulants and illicit use of stimulants for cognitive enhancement in pupils and students in Germany. Pharmacopsychiatry. 2011;44: 60–66. doi: 10.1055/s-0030-1268417 21161883 10.1055/s-0030-1268417

[56] Teter CJ, McCabe SE, LaGrange K, Cranford JA, Boyd CJ. Illicit use of specific prescription stimulants among college students: prevalence, motives, and routes of administration. Pharmacotherapy. 2006;26: 1501–1510. doi: 10.1592/phco.26.10.1501 16999660 10.1592/phco.26.10.1501 PMC1794223

[57] Hilbig BE, Zettler I. When the cat's away, some mice will play: A basic trait account of dishonest behavior. J Res Pers. 2015;57: 72–88.

