# Correction: Physical and cognitive doping in university students using the unrelated question model (UQM): Assessing the influence of the probability of receiving the sensitive question on prevalence estimation

**DOI:** 10.1371/journal.pone.0258705

**Published:** 2021-10-12

**Authors:** Pavel Dietz, Anne Quermann, Mireille Nicoline Maria van Poppel, Heiko Striegel, Hannes Schröter, Rolf Ulrich, Perikles Simon

There is an error in the fourth sentence of the second paragraph of the Statistics subsection of the Materials and methods section. The correct sentence is: Thus, one may assume a prevalence estimate of 0.23, 0.31, or 0.40 under *p* ≈ 1/3.
