# Supplementary material for: Physical and cognitive doping in university students using the unrelated question model (UQM): Assessing the influence of the probability of receiving the sensitive question on prevalence estimation
Source: PLoS One. 2018 May 15;13(5):e0197270. doi: 10.1371/journal.pone.0197270 (PMC5953456; doi:10.1371/journal.pone.0197270)
Supplement: S1 Appendix — (DOCX) [file pone.0197270.s001.docx]

**S1 Appendix**

Let $P(A|Y)$ be the probability that a participant has the stigmatizing attribute $A$ given this participant gives an affirmative response $Y$ (i.e., a “yes”-response). This appendix shows how this probability depends on the parameters$p$, $\pi_{s},$ and$\pi_{n}$. According to Bayes’s rule, this probability is given by

$P(A|Y)=\frac{P(Y|A)\cdot P(A)}{P(Y|A)\cdot P(A)+P(Y|\bar{A})\cdot P(\bar{A})}$,

where $P(Y|A)$ denotes the conditional probability that a participant with the stigmatizing attribute $A$ gives an affirmative response when he or she follows the instruction of UQM. It can easily be seen that this probability is

$P(Y|A)=p+(1-p)\cdot\pi_{n}$.

Furthermore, $P(Y|\bar{A})$ is the conditional probability that a participant without the stigmatizing attributes gives an affirmative response when he or she follows the instruction, and this probability is

$P(Y|\bar{A})=(1-p)\cdot\pi_{n}$.

Combining these results yields,

$P(A|Y)=\frac{[p+(1-p)\cdot\pi_{n}]\cdot\pi_{s}}{[p+(1-p)\cdot\pi_{n}]\cdot\pi_{s}+(1-p)\cdot\pi_{n}\cdot(1-\pi_{s})}$.

The figure below illustrates this equation. It can be seen that the conditional probability $P(A|Y)$ increases monotonically as $p$ increases. In other words, an affirmative response $Y$ rather suggests that a participant possess the stigmatizing attribute $A$ when $p$ is large that when $p$ is small. Thus is quite possible that participants with a stigmatizing attributes tend to provide a dishonest “no”-response, when $p$ is large. The present study tests this prediction.


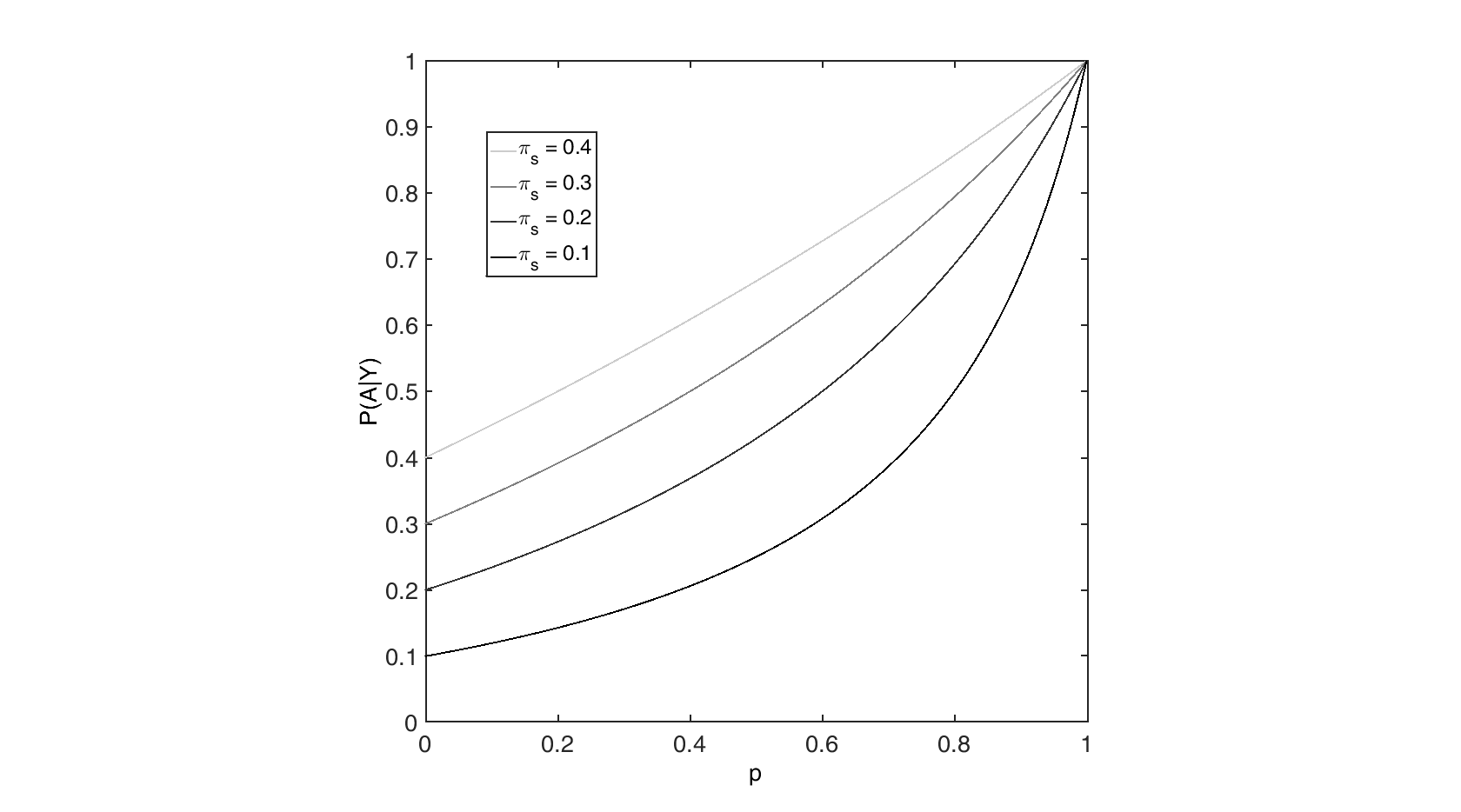


**Figure Conditional probability $P(A|Y)$ as a function of $p$ and $\pi_{s}$**
